# Toxicity of TiO_2_ Nanoparticles to *Escherichia coli*: Effects of Particle Size, Crystal Phase and Water Chemistry

**DOI:** 10.1371/journal.pone.0110247

**Published:** 2014-10-13

**Authors:** Xiuchun Lin, Jingyi Li, Si Ma, Gesheng Liu, Kun Yang, Meiping Tong, Daohui Lin

**Affiliations:** 1 College of Environmental and Biological Engineering, Putian University, Fujian, China; 2 Department of Environmental Science, Zhejiang University, Hangzhou, China; 3 Zhejiang Provincial Key Laboratory of Organic Pollution Process and Control, Zhejiang University, Hangzhou, China; 4 College of Environmental Sciences and Engineering, Peking University, Beijing, P. R. China; Argonne National Laboratory, United States of America

## Abstract

Controversial and inconsistent results on the eco-toxicity of TiO_2_ nanoparticles (NPs) are commonly found in recorded studies and more experimental works are therefore warranted to elucidate the nanotoxicity and its underlying precise mechanisms. Toxicities of five types of TiO_2_ NPs with different particle sizes (10∼50 nm) and crystal phases were investigated using *Escherichia coli* as a test organism. The effect of water chemistry on the nanotoxicity was also examined. The antibacterial effects of TiO_2_ NPs as revealed by dose-effect experiments decreased with increasing particle size and rutile content of the TiO_2_ NPs. More bacteria could survive at higher solution pH (5.0–10.0) and ionic strength (50–200 mg L^−1^ NaCl) as affected by the anatase TiO_2_ NPs. The TiO_2_ NPs with anatase crystal structure and smaller particle size produced higher content of intracellular reactive oxygen species and malondialdehyde, in line with their greater antibacterial effect. Transmission electron microscopic observations showed the concentration buildup of the anatase TiO_2_ NPs especially those with smaller particle sizes on the cell surfaces, leading to membrane damage and internalization. These research results will shed new light on the understanding of ecological effects of TiO_2_ NPs.

## Introduction

Due to their unique chemical and physical properties, titanium dioxide (TiO_2_) nanoparticles (NPs) are produced at a large scale for industrial applications to meet with ever-increasing market demands [1]. The annual production of TiO_2_ NPs is predicted to reach 2.5 million tons by 2025 [2]. The widely used TiO_2_ NPs would find their way into aquatic environments [3]–[6] and interact with aquatic organisms [7]. Eco-toxicity of TiO_2_ NPs is therefore received worldwide research attentions [8]–[16].

Bacteria, e.g., *Escherichia coli* (*E.coli*), as single cell organisms and ubiquitous in aquatic environments, are good model organisms for studying the eco-toxicity of NPs and the cell/organism-NP interaction. Many research works [8] have investigated the toxicity of various TiO_2_ NPs toward *E.coli*, with a focus on the influencing factors such as: (1) Size. Many studies attributed the toxicity of TiO_2_ NPs to their small particle size [17]–[22]. (2) Crystal structure. It is generally concluded that anatase TiO_2_ NPs are more toxic than rutile NPs by inducing greater oxidative stress [15], [23], [24]. (3) Experimental matrix. Changes in water chemistry (e.g., pH and ionic strength) may influence the agglomeration and sedimentation characteristics of NPs and then their toxicity [12], [21], [25]–[28]. (4) Solar radiation, especially those in the UVA region, is also considered as a critical factor of aquatic nanotoxicity [13], [29]–[35]. These researches substantially increased our knowledge on the eco-toxicity of TiO_2_ NPs.

However, controversial and inconsistent results on the toxicity of TiO_2_ NPs are commonly found in recorded studies and precise mechanisms of the nanotoxicity warrant more specific researches. For example, Adams et al. (2006) reported 44% reduction in the growth of *E.coli* by 1 g L^−1^ and 72% reduction by 5 g L^−1^ TiO_2_ NPs (66 nm, crystal structure not determined) [36]; Tong et al. (2013) [21] reported 70% reduction in the growth of *E.coli* by 10 mg L^−1^ TiO_2_ NPs while 30% reduction was observed by Planchon et al. (2013) [15] with the same TiO_2_ NPs at 10 mg L^−1^ (P25, consisting of an 80∶20 ratio of anatase:rutile). So an acute lack of emphasis on the environmental and nanoparticle parameters prevents a meaningful comparative assessment from the hitherto available nanotoxicity data, and it highlights the necessity to provide additional eco-toxicological studies and physicochemical characterization of TiO_2_ NPs to ensure consistency of research results.

This study is aimed to elucidate the roles of particle size and crystal structure in the toxicity of TiO_2_ NPs using *E.coli* as a model organism. Five types of well characterized TiO_2_ NPs with different particle sizes and crystal phases were examined. The toxicity assays were conducted at different concentrations of the NPs and various solution pHs and ionic strengths. In addition, the interactions between TiO_2_ NPs and bacteria and the cell reactive responses were examined with transmission electron microscopy (TEM) and measurements of intracellular reactive oxygen species (ROS) and malondialdehyde (MDA) to address the toxicity mechanism. The results are believed to increase our understanding of the nanotoxicology.

## Materials and Methods

### 1. Nanoparticles and characterizations

Five types of TiO_2_ NPs were purchased and used in this study. They were anatase TiO_2_ with particle sizes measured to be around 10 nm (TiO_2_-NP 10A), 25 nm (TiO_2_-NP 25A), and 50 nm (TiO_2_-NP 50A) and rutile TiO_2_ of 50 nm (TiO_2_-NP 50R) and mixed anatase and rutile TiO_2_ of 25 nm (TiO_2_-NP25 AR). TiO_2_-NP 10A and TiO_2_-NP 50R were from Hongsheng Material Sci & Tech Co., Zhejiang, China and the other three TiO_2_ NPs from Wangjing New Material Sci & Tech Co., Zhejiang, China.

Morphologies of the NPs were examined using TEM (JEM-1230, JEOL Ltd., Tokyo, Japan). Powered X-ray diffraction analysis (XRD, X’Pert Pro, Holland) was carried out to characterize the crystal structure of the NPs. Elemental compositions of the NPs were determined by using an X-ray energy dispersion spectroscope (EDS, GEN-ESIS 4000, EDAX Inc. America). Hydrodynamic diameters and zeta potentials of the NPs (50 mg L^−1^) were measured with a Zetasizer (Nano ZS90, Malvern, UK) after being sonicated (100 W, 40 kHz, 30 min) into 100 mg L^−1^ NaCl solution at 25°C and various pH values. Points of zero charge (pH_pzc_) of the NPs were obtained from the zeta potential versus pH curves. Specific surface areas of the NPs were determined using the multi-point Brunauer-Emmett-Teller (BET) method (Quantachrome NOVA 2000e, America).

### 2. Dose-effect experiments

*E.coli* O111 (Genbank access no. GU237022.1) isolated from a sewage water was used as the test organism, as reported in our previous studies [37], [38]. The bacteria were maintained in Luria Bertani (LB) solid plates at 4°C and inoculated in LB broth (pH 7.2∼7.4) at 37°C overnight (12∼16 h) at 150 rpm. The bacteria were separated from the broth by centrifugation at 3000 *g* for 15 min and washed twice with 0.85% physiology salt-water. The bacterium stock suspension was prepared by resuspending the bacterial pellets in 0.85% NaCl physiology salt-water with the cell concentration determined by the absorbance at 600 nm (OD_600_) being adjusted to 1.0.

The stock NP suspensions (500 mg L^−1^) were obtained by sonicating (100 W, 40 kHz, 30 min) 50 mg of the TiO_2_ NPs into 100 mL of ultra-pure water. The stock NP suspensions after sonication were diluted using ultra-pure water to the target test concentrations (10–500 mg L^−1^).

One mL of the *E.coli* stock suspension was added into 100 mL of the test NP suspensions. The mixtures were placed on a shaker at 37°C and 150 rpm for 3 h with natural light. The bacteria in the resultant mixtures were spread on LB agar plates and incubated at 37°C for 24 h, and the colonies were counted. The percentage viabilities of the bacteria in the NP suspensions were calculated by dividing their colony forming units (CFU) mL^−1^ by that in the NP-free control. All treatments including the control were repeated in triplicate.

### 3. TEM Observations

TEM was used to observe the direct contact between the NPs and the bacterial cells. A drop of the bacteria exposed to the NPs (50 mg L^−1^, 3 h) and the NP-free control was air-dried onto a copper grid and was then imaged by the TEM. To observe the internalization and localization of the NPs in the cells and the changes in cellular structure as affected by the NPs, the NPs-treated and untreated bacteria were fixed in 2.5% glutaraldehyde, dehydrated in graded concentrations of ethanol, embedded in Epon resin, and stained with OsO_4_ [12], [39]. Ultrathin sections were then cut and counterstained with Reynold’s and uranyl acetate for the TEM observation.

### 4. Reactive oxygen species (ROS) and lipid peroxidation measurements

The fluorescence probe 2′7′-dichlorodihydrofluorescein diacetate (H_2_DCFDA) was used to quantify the formation of intracellular ROS as described in our previous papers [12], [39] with minor modifications. The bacterial cells after exposure to the test media were collected by centrifugation (8000 *g*, 5 min). The pellet was resuspended in 0.85% physiology salt-water containing 10 µM H_2_DCFDA and incubated on the shaker (150 rpm) for 30 min at 37°C. The bacteria were further pelleted and resuspended in 300 µL of 0.85% physiology salt-water. The fluorescence values were measured in a 96-well plate using a multifunctional microplate reader (M200 PRO, Ltd., Austria) with the excitation and emission wavelengths of 485 nm and 528 nm, respectively. Relative ROS levels were calculated by the fluorescence ratio of the treatments to the control.

The concentration of malondialdehyde (MDA) was determined as an indicator of lipid peroxidation as described in previous works [12], [39]. Briefly, the exposed bacteria were mixed into 1 mL of 10% (wt/vol) trichloroacetic acid and left at room temperature for 10 min; the supernatant of the mixture was collected after centrifugation at 11,000 *g* for 40 min and then mixed with 1.5 mL of a freshly prepared 0.67% (wt/vol) thiobarbituric acid solution; the resultant mixture was incubated for 20 min in a boiling water bath and after cooling the absorbance was measured at 532 nm; and the MDA concentration was calculated using the Hodges’ equations.

### 5. Effects of pH and ionic strength

TiO_2_-NP 10A with a concentration fixed at 10 mg L^−1^ was selected as a type of representative NPs in examining the effect of water chemistry on the nanotoxicity. In the pH effect experiment, the suspension pHs were adjusted to 5.0, 7.0, 8.0 and 10.0 using 0.1 M HCl and NaOH; NaCl was added to maintain a constant background ionic strength (100 mg L^−1^). For the ionic strength effect experiment, difference concentrations of NaCl (0, 50, 100, 150 and 200 mg L^−1^) were added into the NP suspensions; the final suspension pH remained at about neutral without further adjustment. The bacterial exposure method in the pH and ionic strength effect experiments was the same as the above dose-effect experiments. Zeta potential of 1 mL of the stock bacterial suspension after being mixed into 100 mL of ultra-pure water at pH 5.0, 7.0, 8.0 and 10.0 was also measured by the Zetasizer.

### 6. Statistical analysis

Each treatment including the blank control was conducted in triplicate and the results were presented as mean ± SD (standard deviation). For each datum point, data were normalized by the reference without NPs. This representation thus strictly reports the incremental impacts of TiO_2_ NPs, excluding the medium stress. The Student’s *t* test was performed to analyze the significance of difference between two groups of data. Origin 8.0 was used to make graphs. The concentration resulting in 50% mortality (LC_50_) was calculated with SPSS 20.0.

## Results and Discussion

### 1. Characteristics of TiO_2_ NPs

**Figure 1:**
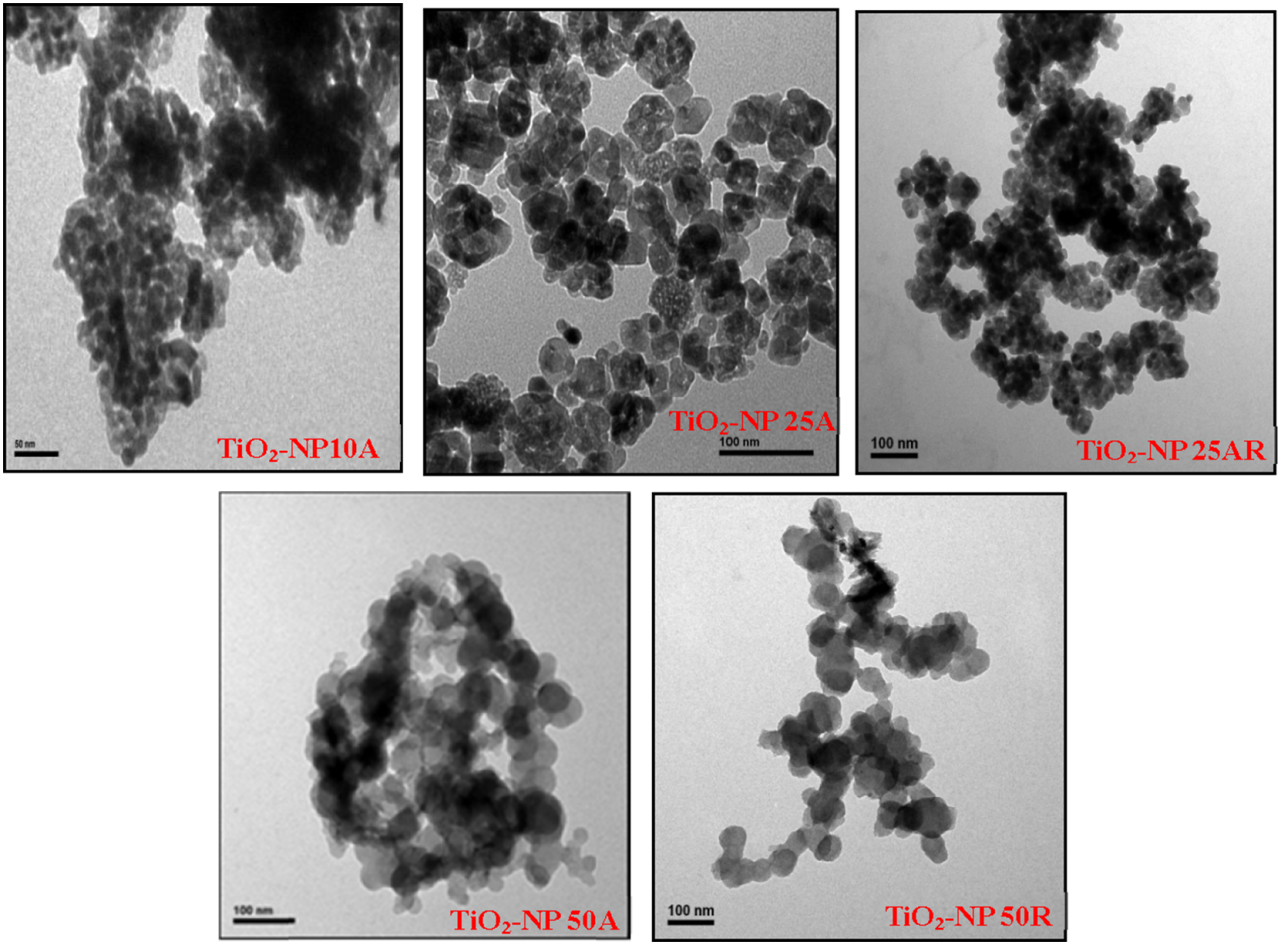
TEM images of the as-received TiO_2_ NPs.

**Figure 2:**
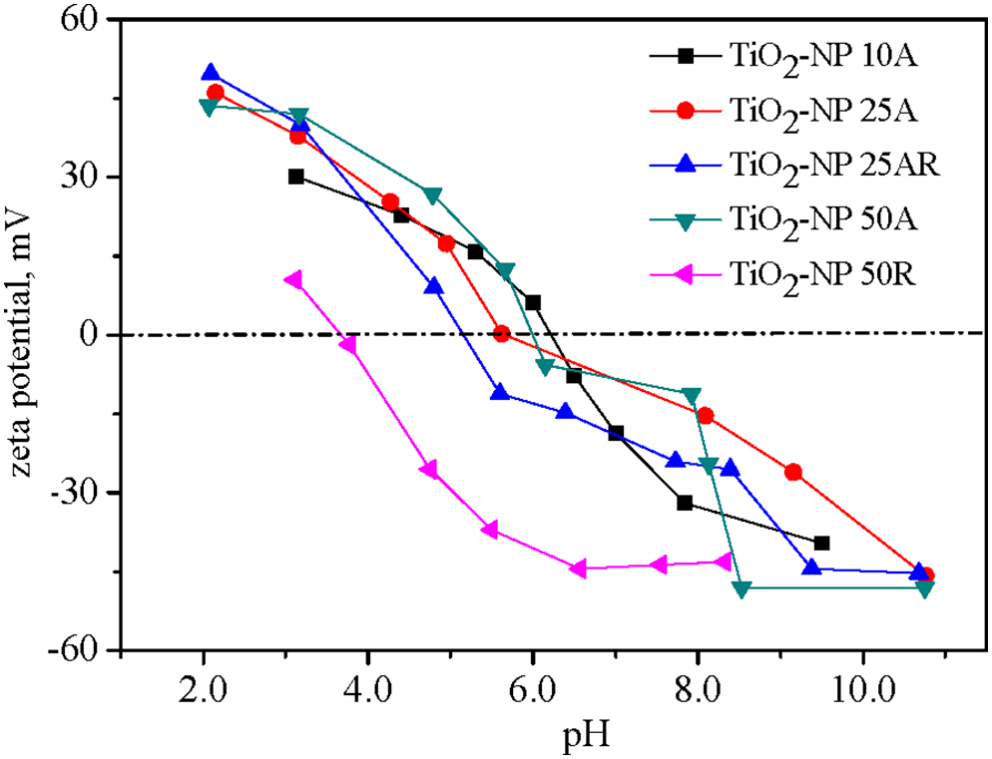
Changes of zeta potentials of the TiO_2_ NPs against a solution pH.

**Figure 3:**
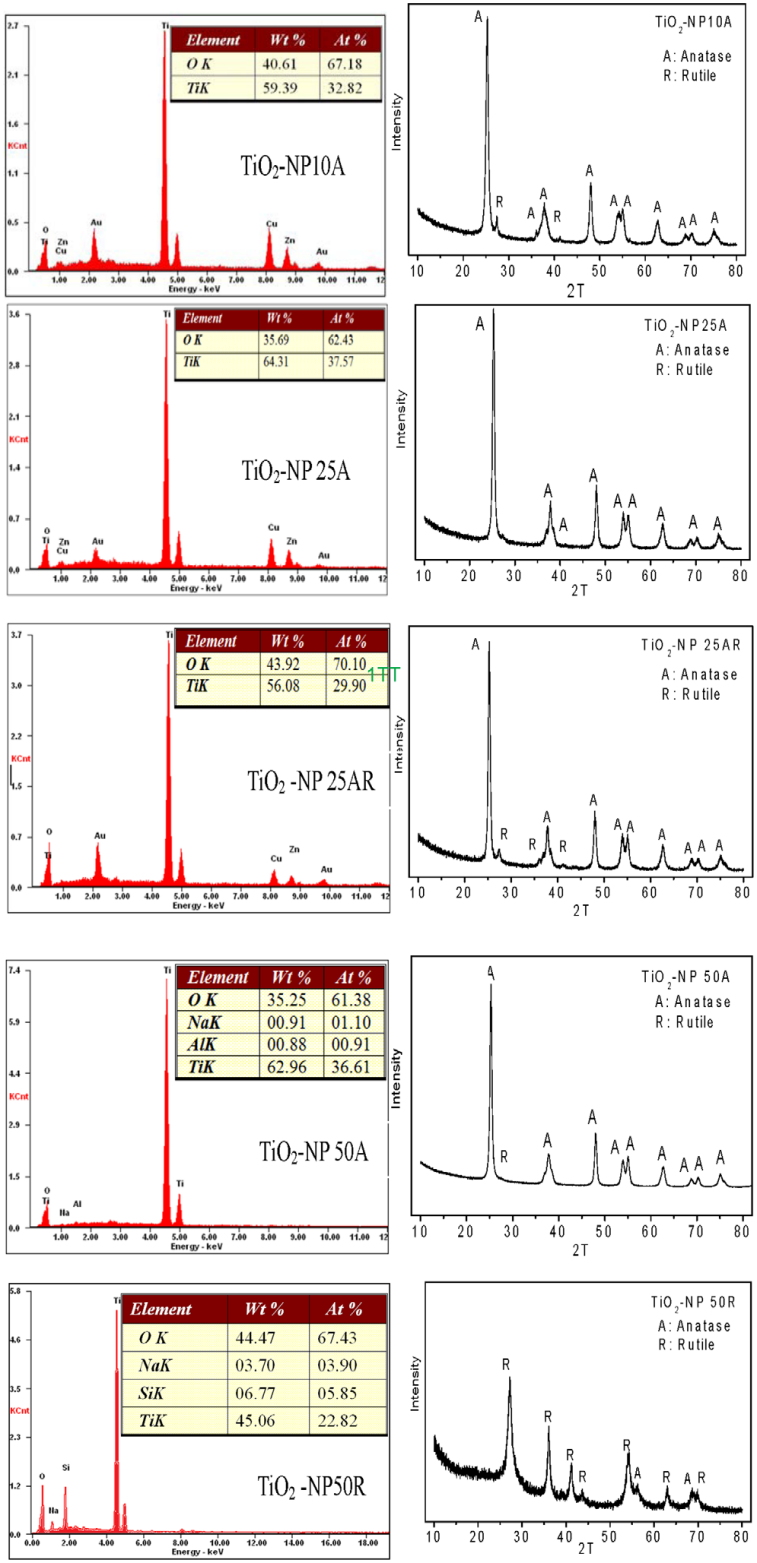
EDS (left column) and XRD (right column) figures of the as-received TiO_2_ NPs.

Selected properties of the TiO_2_ NPs are listed in Table 1 with their TEM images shown in Figure 1. Big NP aggregates present in the TEM images and the measured large hydrodynamic diameters indicate the aggregation of the NP suspensions even after the sonication. TiO_2_-NP 10A, having the smallest particle size (TEM size of 11.0±3.4 nm) among the five TiO_2_ NPs, owned relative greater hydrodynamic diameter of 314±8 nm. Changes in zeta potentials of the TiO_2_ NPs against a solution pH are shown in Figure 2. The calculated pH_pzc_ of the NPs varied from pH 3.6 to 6.2, which could account for their negative zeta potentials (−33.8– −5.48 mV) in the neutral toxicity test medium. Specific surface areas of the TiO_2_ NP_S_ ranged from 30 m^2 ^g^−1^ (TiO_2_-NP 50R) to 324 m^2 ^g^−1^ (TiO_2_-NP 10A). Purities of the NPs determined by the EDS were all above 98.0% (Figure 3). XRD patterns of the TiO_2_ NP_S_ (Figure 3) confirmed the predominance of anatase phase of TiO_2_-NP 10A, TiO_2_-NP 25A, and TiO_2_-NP 50A and the rutile nature of TiO_2_-NP 50R; and TiO_2_-NP 25AR was a mixture of anatase (93%) and rutile (7%).

**Table 1:** Characteristics of the nanoparticles.

Sample	Crystalphase, %	Zeta potential, mV	pH_pzc_	hydrodynamic size, nm	S_BET_, m^2 ^g^−1^	TEM size, nm
TiO_2_-NP10A	Anatase,100	−21.6	6.2	314±8	324	11.0±3.4
TiO_2_-NP25A	Anatase,99.2	−5.48	5.6	251±37	77	26.2±6.1
TiO_2_-NP25AR	Anatase,93.0	−13.6	5.2	202±57	66	26.7±5.0
TiO_2_-NP50A	Anatase,98.8	−9.34	6.0	486±12	105	57.1±14.0
TiO_2_-NP50R	Rutile, 100	−33.8	3.6	260±10	30	57.2±17.8

Note: the crystal phase was determined by the XRD; zeta potential and hydrodynamic size were measured in the toxicity test medium at pH 6.5 by the Zetasizer; pH_pzc_ was calculated from the zeta potential versus pH curves shown in Figure 2; S_BET_ (specific surface area) measured using the BET (Brunauer-Emmett-Teller) method; TEM size shows the NP size measured with the TEM images.

### 2. Cell viability assessment

**Figure 4:**
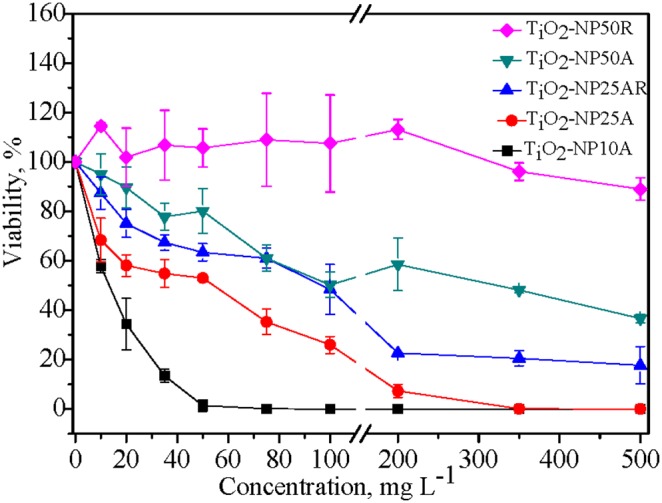
Variations of the bacteria viability with concentrations of the TiO_2_ NPs. The viability was the ratio of bacterial cell number under the NP treatment to the blank control.

The particle dose, size and phase dependent reductions in the cell viability of *E.coli* upon exposure to the TiO_2_ NPs for 3 h were observed through the plate count assay (Figure 4). The four anatase NPs were more or less toxic to *E.coli* and the viability of the bacteria exhibited a pronounced concentration-dependent decrease. The calculated 3 h LC_50_ of the four anatase NPs had an order of TiO_2_-NP 10A (17.0 mg L^−1^)<TiO_2_-NP 25A (59.2 mg L^−1^)<TiO_2_-NP 25AR (163 mg L^−1^)<TiO_2_-NP 50A (304 mg L^−1^). The enhancement of bactericidal effect of the NPs with decreasing particle size was observed throughout the various particle concentrations. TiO_2_-NP 10A in the anatase phase with the minimum particle size and the largest BET surface area was determined to be the most toxic to *E.coli*. The presence of rutile phase in the NPs lowered the bactericidal activity in comparison to the pure anatase NPs. As shown in Figure 4, although similar in particle size, the toxicity of TiO_2_-NP 25AR was much lower than that of TiO_2_-NP 25A; the pure rutile TiO_2_-NP 50R was nontoxic to the bacteria with concentration up to 500 mg L^−1^, while the anatase TiO_2_-NP 50A could inactivate half of the bacteria at 304 mg L^−1^.

### 3. TEM observations of the direct NP-cell interactions

**Figure 5:**
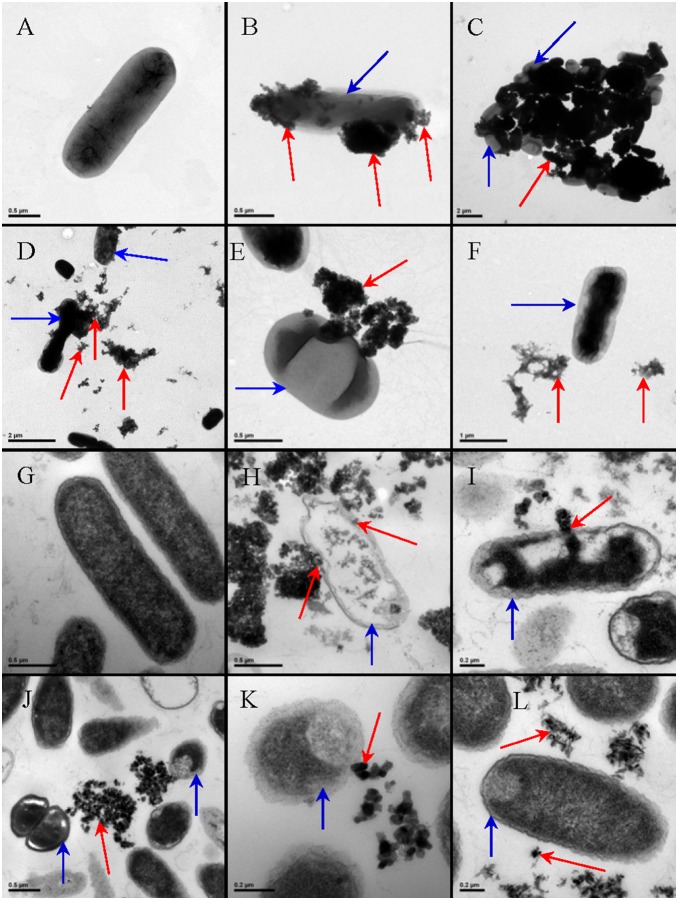
Selected TEM images of the unsliced (A to F) and sliced (G to L) *E.coli* cells without (A and G) and with the treatments of TiO_2_-NP 10A (B and H), TiO_2_-NP 25A (C and I), TiO_2_-NP 25AR (D and J), TO_2_-NP 50A (E and K) and TiO_2_-NP 50R (F and L). The blue arrows point to the cells and the red arrows direct to the NP aggregates.

Nanoparticle-type-dependent bacterial cell membrane localizations of the TiO_2_ NPs as well as morphological changes of the NPs-exposed cells were captured by the TEM images (Figure 5). The stronger NP-cell interaction was observed for the TiO_2_ NPs with anatase crystal structure and smaller particle size. Numerous TiO_2_-NP 10A aggregates with various sizes were observed tightly attached to the bacterial cell surfaces (Figure 5B). The big and tight NP-cell aggregate in Figure 5C indicates the strong interaction between TiO_2_-NP 25A and the cells. Some of the TiO_2_-NP 25AR aggregates were also observed attaching to the bacterial cells but some present away from the cells (Figure 5D), implying the relatively weaker NP-cell interaction as compared with the pure anatase TiO_2_-NP 25A of the same size. A few big aggregates of TiO_2_-NP 50A were observed loosely attached to the bacterial cell (Figure 5E), which suggests the much weaker interaction of TiO_2_-NP50A than the smaller sized TiO_2_-NP 25A and TiO_2_-NP 10A with the cells. No obvious attachment between the TiO_2_-NP 50R aggregates and the bacterial cells was observed (Figure 5F).

Figures 5G to 5L show TEM images of the sliced bacterial cells untreated or treated with 50 mg L^−1^ of the TiO_2_ NPs. The untreated (Figure 5G) and TiO_2_-NP 50R-treated (Figure 5L) cells remained intact with unimpaired cell morphology and structure, indicating the nontoxicity of TiO_2_-NP 50R. However, the NPs with smaller size and anatase phase were observed sticking to the cell surfaces (Figure 5H to 5K), which apparently induced cell distortion, plasmolysis and cell wall and membrane damage; penetration and internalization of the nanoparticles into the bacterial cells were also observed (Fig. 5H and 5I).

From the above TEM observations, it can be concluded that anatase TiO_2_ NPs are more prone to attaching on the bacterial surfaces than rutile NPs, and the larger NPs interact weaker with cells compared to the smaller NPs. As particle size decreases, the ratio of surface area to mass increases and changes in the physicochemical properties (e.g., surface atom reactivity, electronic and optical properties) of the nanoparticles occur, consequently, the smaller particles tend to agglomerate to a greater extent, which can further influence their reactivity and binding characteristics [40]. The NP-cell attachment may inhibit the movement of substances in and out of bacterial cells, thereby causing homeostatic imbalance, cellular metabolic disturbance and even cell death [41]. Moreover, the NP-cell attachment would facilitate the cell internalization of NPs and the intracellular ROS production [12]. If TiO_2_ NPs are sufficiently small, they can penetrate in the cells, and then induce the potential photocatalytic process inside and adsorb and deactivate biomolecules such as proteins [22], [42]. Therefore, the physical NP-cell attachment and interaction could substantially contribute to the observed nanotoxicity. Many studies [13], [43]–[44] suggest that the antibacterial mechanism of NPs includes the disruption of bacterial cellular membrane. However, we do not know for sure yet why the anatase NPs had higher affinity to the cell surfaces than the rutile NPs, which could be possibly due to their different surface properties. It is indicated that the coordination and surface properties allow anatase but not rutile NPs after dispersion induce the generation of ROS [24].

### 4. Oxidative stress and lipid peroxidation induced by the NPs

**Figure 6:**
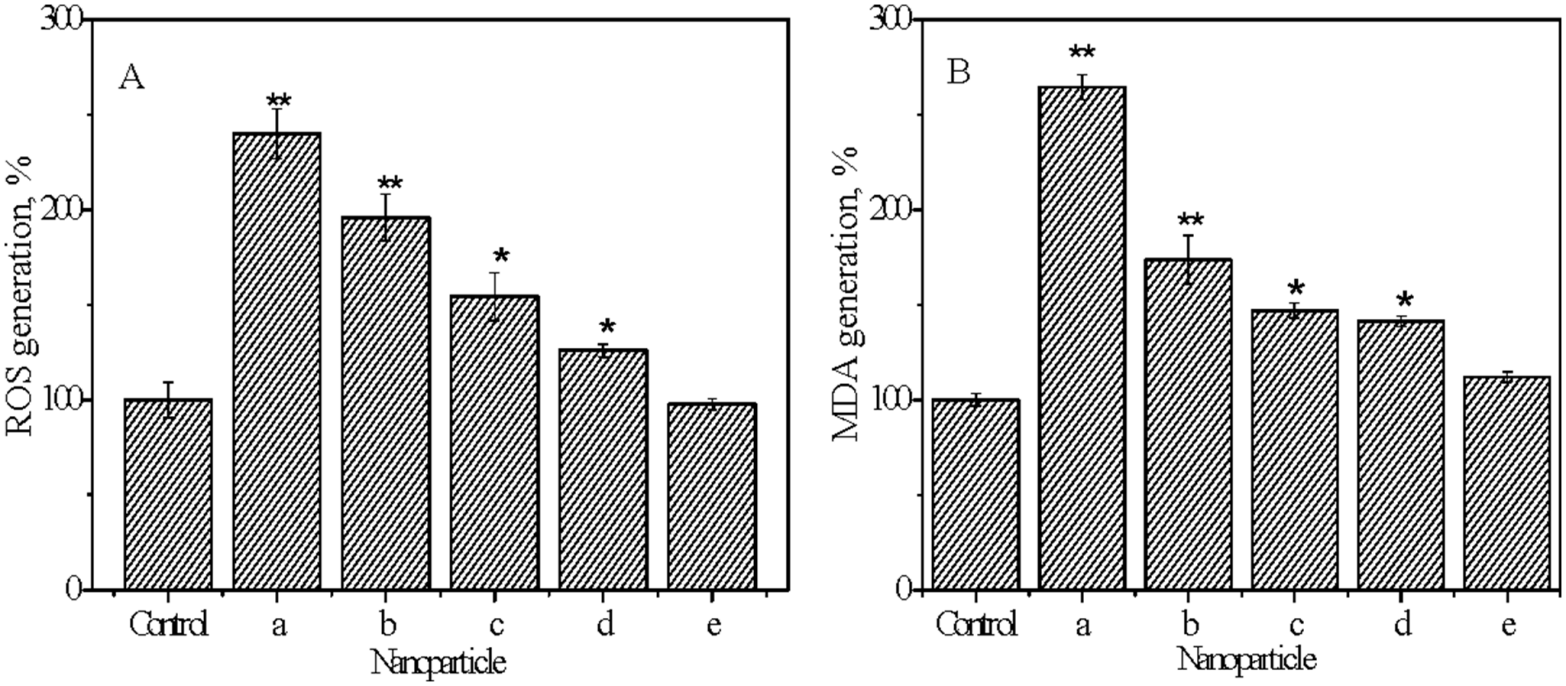
Relative contents of intracellular ROS (A) and MDA (B) in the bacterial cells after 3 h exposure to the TiO_2_ NPs (50 mg L^−1^). a–e stand for TiO_2_-NP 10A, TiO_2_-NP 25A, TiO_2_-NP 25AR, TiO_2_-NP 50A, and TiO_2_-NP 50R, respectively. Asterisk indicates a significant difference relative to the control (*, *p*<0.05; **, *p*<0.01) based on the Student’s *t* test. Error bars represent standard deviation (n = 3).

Relative intracellular ROS productions following the exposures to the TiO_2_ NPs (50 mg L^−1^) are shown in Figure 6A. The produced ROS in the bacterial cells exposed to the anatase TiO_2_ NPs was significantly (p<0.05) higher than that in the blank control cells and increased with decreasing particle size; whereas the pure rutile TiO_2_-NP 50R had insignificant effect on the intracellular ROS production and TiO_2_-NP 25AR containing 7% rutile induced significantly lower intracellular ROS production compared with the pure anatase TiO_2_-NP 25A of the same particle size. The enhanced intracellular ROS would affect protein expression and function in the bacteria by interrupting translation and post-translational modification [45]. It has been indicated that TiO_2_ NPs in anatase phase are capable of inducing generation of more ROS than that in the rutile phase [24], [46] and thereby may cause higher cytotoxicity [47] including toward *E.coli* [13], [48]–[52]. The increased ROS generation in *E.coli* exposed to the smaller and anatase TiO_2_ NPs coincided with their enhanced bactericidal effects as shown in Figure 4, which suggests that the size and crystal phase of TiO_2_ NPs played a critical role in the nanotoxicity and the nanotoxicity could be caused mainly by the elevated oxidative stress.

MDA productions in the *E coli* cells upon the exposures to the 50 mg L^−1^ TiO_2_ NPs are shown in Figure 6B. Significantly higher MDA contents were observed in the NPs-treated cells compared with the blank control, indicating the cell membrane lipid peroxidation induced by the NPs. The MDA content was the highest in the TiO_2_-NP 10A treated cells and overall decreased with decreasing size of the anatase NPs, which was in the same order of the ROS production by anatase NPs. This implies that the lipid peroxidation could be mainly caused by the increased ROS.

### 5. Effects of pH and ionic strength on the nanotoxicity

**Figure 7:**
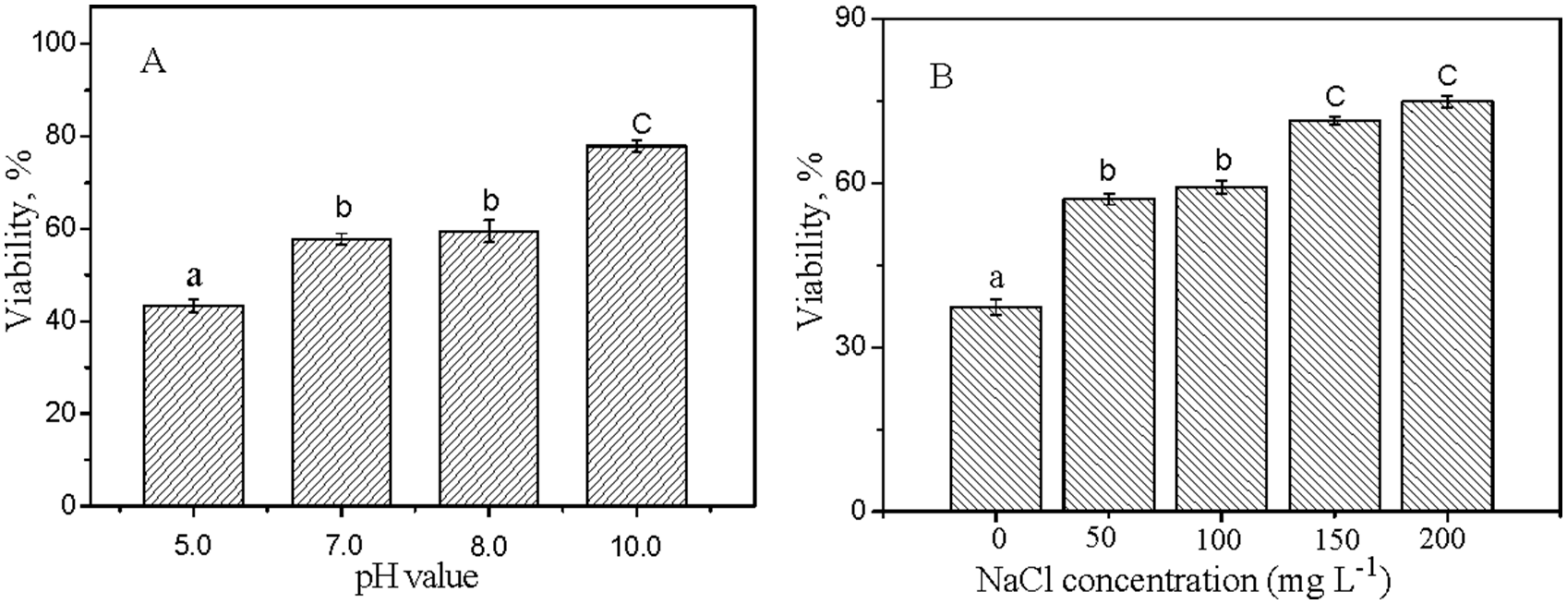
The effects of pH (A) and NaCl (B) on the relative viability of *E.coli* exposed to 10 mg L^−1^ TiO_2_-NP 10A for 3 h. Significant difference (*p*<0.05) between two treatments is presented by different lowercase letters a, b and c. Error bars represent standard deviation (n = 3).

The bactericidal effect of the 10 mg L^−1^ TiO_2_-NP 10A exhibited a significant dependence on the solution chemistry (Figure 7). No significant difference in the nanotoxicity was observed between pH 7.0 and 8.0, while the exposed bacteria presented significantly lower and higher viability at pH 5.0 and 10.0 as compared with that at pH 7.0, respectively (Figure 7A). Increasing the suspension pH from 5.0 to 10.0, the bacterial viability increased from 43.3% to 77.9%, indicating the decreasing nanotoxicity with increasing pH. It is generally considered that direct contact and adherence of NPs with the organism cell surfaces plays a critical role in the nanotoxicity [12]. Zeta potentials of the bacterial cells were all negative at the four pHs, being −58.4, −56.7, −56.7 and −52.5 mV at pH 5.0, 7.0, 8.0 and 10.0, respectively; whereas zeta potential of TiO_2_-NP 10A decreased from about 20 mV at pH 5.0 to lower than −40 mV at pH 10.0 (Figure 2). The positively-charged NPs at pH 5.0 could have a higher potential of contact and hetero-agglomeration with the negatively-charged bacterial cells through the electrostatic attraction and therefore had a higher antibacterial effect compared with the negatively-charged NPs at the three higher pHs.

It has been indicated that the antibacterial effect of TiO_2_ NPs (25 nm, P25) on *E.coli* was stronger at pH 5.5 versus 7.0 and 9.5 and the stronger antibacterial effect at the lower pH was attributed to the stronger accumulation of the NPs on the cell surfaces [53], [54]. However, contradictory research result has also been reported. Planchon et al. (2013) found a stronger adsorption of TiO_2_ NPs (25 nm, P25) on *E.coli* but a slightly lower toxicity at pH 5.0 versus 8.0, which was attributed to a better physiological state of *E.coli* bacteria at pH 5.0 (artificial water) versus 8.0 (surface water sample) [15]. Rincon and Pulgarin (2004) did not observe any difference in the *E.coli* deactivation rate by the TiO_2_ NPs (25 nm, P25) in the pH range of 4.0–9.0 [55]. The contradictory results on the pH effect may partly come from the difference in the crystal structure of the used TiO_2_ NPs, but the exact mechanisms remain to be studied.

It is observed that the addition of NaCl (50∼200 mg L^−1^) reduced the toxicity of TiO_2_-NP 10A toward *E.coli* to an extremely significant extent (Figure 7B). Some studies suggest that NaCl introduced to the medium can decrease the toxicity effect on the bacteria by providing a barrier of steric hindrance between NPs and cells [15]. Li et al. (2013) found that saline ions promoted NP aggregation and reduced surface charge, and then inhibited the adsorption of NPs on bacterial surfaces, so higher saline ions could lead to higher cell viability [38]. Furthermore, ionic strength can also influence the tolerance of bacteria to toxicants [37], [38]. The ionic strength of physiology salt-water (8.5 g L^−1^ NaCl) is isotonic and favorable for the bacterium survival. Hence, the bacteria were more tolerant to the NP suspensions at the higher ionic strengths (closer to the physiology salt-water).

## Conclusions

The present study investigated the antibacterial effect of five types of TiO_2_ NPs with various crystal phase and particle size. A marked particle size and crystal phase dependent nanotoxicity was observed. Water chemistry, i.e. pH and ionic strength, could also significantly influence the bactericidal activity of the anatase TiO_2_ NPs. In conclusion, the TiO_2_ NPs with anatase phase and smaller particle size had higher affinity to the cell surfaces and induced heavier oxidative damage and toxicity to the bacterial cells, and the toxicity decreased with increasing pH (5.0–10.0) and ionic strength (50–200 mg L^−1^ NaCl). These findings substantiate the need to correlate the NP characterization and behavior in environmental matrices with the toxicological endpoints and to develop a common test strategy for the eco-toxicity study of NPs taking into consideration of various confounding factors relating to the NPs, bacterial cells, and the test environment in the near future.

## References

[1] Chen XB, Mao SS (2007) Titanium dioxide nanomaterials: synthesis, properties, modifications, and applications. Chem Rev 107: 2891–2959. 17590053 10.1021/cr0500535

[2] Robichaud CO, Uyar AE, Darby MR, Zucker LG, Wiesner MR (2009) Estimates of upper bounds and trends in nano-TiO_2_ production as a basis for exposure assessment. Environ Sci Technol 43: 4227–4233. 19603627 10.1021/es8032549

[3] Kaegi R, Ulrich A, Sinnet B, Vonbank R, Wichser A, et al (2008) Synthetic TiO_2_ nanoparticle emission from exterior facades into the aquatic environment. Environ Pollut 156: 233–239. 18824285 10.1016/j.envpol.2008.08.004

[4] Lin DH, Tian XL, Wu FC, Xing BS (2010) Fate and transport of engineered nanomaterials in the environment. J Environ Qual 39: 1896–1908. 21284287 10.2134/jeq2009.0423

[5] Johnson AC, Bowes MJ, Crossley A, Jarviea HP, Jurkschat K, et al (2011) An assessment of the fate, behaviour and environmental risk associated with sunscreen TiO_2_ nanoparticles in UK field scenarios. Sci Total Environ 409: 2503–2510. 21501856 10.1016/j.scitotenv.2011.03.040

[6] Batley GE, Kirby JK, Mclaughlin MJ (2013) Fate and risks of nanomaterials in aquatic and terrestrial environments. Accounts Chem Res 46: 854–862. 10.1021/ar2003368 22759090

[7] Ma S, Lin DH (2013) The biophysicochemical interactions at the interfaces between nanoparticles and aquatic organisms: adsorption and internalization. Environ Sci: Processes Impacts 15: 145–160. 10.1039/c2em30637a 24592433

[8] Menard A, Drobne D, Jemec A (2011) Ecotoxicity of nanosized TiO_2_: review of in vivo data. Environ Pollut 159: 677–684. 21186069 10.1016/j.envpol.2010.11.027

[9] Griffitt RJ, Luo J, Gao J, Bonzongo JC, Barber DS (2008) Effects of particle composition and species on toxicity of metallic nanomaterials in aquatic organisms. Environ Toxic Chem 27: 1972–1978. 10.1897/08-002.1 18690762

[10] Battin TJ, Kammer FVD, Weilhartner A, Ottofuelling S, Hofmann T (2009) Nanostructured TiO_2_: transport behavior and effects on aquatic microbial communities under environmental conditions. Environ Sci Technol 43: 8098–8104. 19924929 10.1021/es9017046

[11] Ji J, Long ZF, Lin DH (2011) Toxicity of oxide nanoparticles to the green algae *Chlorella* sp. Chem Eng J 170: 525–530.

[12] Lin DH, Ji J, Long ZF, Yang K, Wu FC (2012) The influence of dissolved and surface-bound humic acid on the toxicity of TiO_2_ nanoparticles to *Chlorella* sp. Water Res 46: 4477–4487. 22704133 10.1016/j.watres.2012.05.035

[13] Dalai S, Pakrashi S, Kumar RSS, Chandrasekaran N, Mukherjee A (2012) A comparative cytotoxicity study of TiO_2_ nanoparticles under light and dark conditions at low exposure concentrations. Toxicol Res 1: 116–130.

[14] Clément L, Hurel C, Marmier N (2013) Toxicity of TiO_2_ nanoparticles to cladocerans, algae, rotifers and plants – effects of size and crystalline structure. Chemosphere 90: 1083–1090. 23062945 10.1016/j.chemosphere.2012.09.013

[15] Planchon M, Ferrari R, Guyot F, Gélabertb A, Menguy N, et al (2013) Interaction between *Escherichia coli* and TiO_2_ nanoparticles in natural and artificial waters. Colloid Surface B 102: 158–164. 10.1016/j.colsurfb.2012.08.034 23006561

[16] Kim J, Lee S, Kim C, Seo J, Park Y, et al (2014) Non-monotonic concentration–response relationship of TiO_2_ nanoparticles in freshwater cladocerans under environmentally relevant UV-A light. Ecotox Environ Safe 101: 240–247. 10.1016/j.ecoenv.2014.01.002 24507152

[17] Jiang JK, Oberdőrster G, Biswas P (2009) Characterization of size, surface charge, and agglomeration state of nanoparticle dispersions for toxicological studies. J Nanopart Res 11: 77–89.

[18] Kim DS, Kwak SY (2009) Photocatalytic inactivation of *E.coli* with a mesoporous TiO_2_ coated film using the film adhesion method. Environ Sci Technol 43: 148–151. 19209598 10.1021/es801029h

[19] Deckers AS, Loo S, L’hermite MM, Boime NH, Menguy N, et al (2009) Size-, composition- and shape-dependent toxicological impact of metal oxide nanoparticles and carbon nanotubes toward bacteria. Environ Sci Technol 43: 8423–8429. 19924979 10.1021/es9016975

[20] Park S, Lee S, Kim B, Lee S, Lee J, et al (2012) Toxic effects of titanium dioxide nanoparticles on microbial activity and metabolic flux. Biotechnol Bioproc E 17: 276–282.

[21] Tong TZ, Binh CTT, Kelly JJ, Gaillard JF, Gray KA (2013) Cytotoxicity of commercial nano-TiO_2_ to *Escherichia coli* assessed by high-throughput screening: effects of environmental factors. Water Res 47: 2352–2362. 23466221 10.1016/j.watres.2013.02.008

[22] Xiong SJ, George SJ, Ji ZX, Lin SJ, Yu HY, et al (2013) Size of TiO_2_ nanoparticles influences their phototoxicity: an in vitro investigation. Arch Toxicol 87: 99–109. 22885792 10.1007/s00204-012-0912-5 PMC5889301

[23] Nel A, Xia T, Mädler L, Li N (2006) Toxic potential of materials at the nanolevel. Science 311: 622–627. 16456071 10.1126/science.1114397

[24] Jin C, Tang Y, Yang FG, Li XL, Xu S, et al (2011) Cellular Toxicity of TiO_2_ nanoparticles in anatase and rutile crystal phase. Biol Trace Elem Res 141: 3–15. 20506001 10.1007/s12011-010-8707-0

[25] Whirter MJM, Quillan AJM, Bremer PJ (2002) Influence of ionic strength and pH on the first 60 min of *Pseudomonas aeruginosa* attachment to ZnSe and to TiO_2_ monitored by ATR-IR spectroscopy. Colloid Surface B 26: 365–372.

[26] French RA, Jacobson AR, Kim B, Isley SL, Penn RL, et al (2009) Influence of ionic strength, pH, and cation valence on aggregation kinetics of titanium dioxide nanoparticles. Environ Sci Technol 43: 1354–1359. 19350903 10.1021/es802628n

[27] Chowdhury I, Cwiertny DM, Walker SL (2012) Combined factors influencing the aggregation and deposition of nano-TiO_2_ in the presence of humic acid and bacteria. Environ Sci Technol 46: 6968–6976. 22455349 10.1021/es2034747

[28] Ng AMC, Chan CMN, Guo MY, Leung YH, Djurišić AB, et al (2013) Antibacterial and photocatalytic activity of TiO_2_ and ZnO nanomaterials in phosphate buffer and saline solution. App Microbiol Biot 97: 5565–5573. 10.1007/s00253-013-4889-7 23661082

[29] Brunet L, Lyon DY, Hotze EM, Alvarez PJJ, Wiesner MR, et al (2009) Comparative photoactivity and antibacterial properties of C_60_ fullerenes and titanium dioxide nanoparticles. Environ Sci Technol 43: 4355–4360. 19603646 10.1021/es803093t

[30] Jiang W, Mashayekhi H, Xing BX (2009) Bacterial toxicity comparison between nano- and micro-scaled oxide particles. Environ Pollut 157: 1619–1625. 19185963 10.1016/j.envpol.2008.12.025

[31] Kim SW, An YJ (2012) Effect of ZnO and TiO_2_ nanoparticles preilluminated with UVA and UVB light on *Escherichia coli* and *Bacillus subtilis* . App Microbiol Biot 95: 243–253. 10.1007/s00253-012-4153-6 22615055

[32] Bokare A, Sanap A, Pai M, Sabharwal S, Athawale AA (2013) Antibacterial activities of Nd doped and Ag coated TiO_2_ nanoparticles under solar light irradiation. Colloid Surface B 102: 273–280. 10.1016/j.colsurfb.2012.08.030 23010118

[33] Li S, Wallis LK, Ma H, Diamond SA (2014) Phototoxicity of TiO_2_ nanoparticles to a freshwater benthic amphipod: Are benthic systems at risk? Sci Total Environ 466–467: 800–808. 10.1016/j.scitotenv.2013.07.059 23973546

[34] Li S, Wallis LK, Diamond SA, Ma H, Hoff DJ (2014) Species sensitivity and dependence on exposure conditions impacting phototoxicity of TiO_2_ nanoparticles to benthic organisms. Environ Toxicol Chem 33: 1563–1569. 24846372 10.1002/etc.2583

[35] Li S, Pan X, Wallis LK, Fan ZY, Chen ZL, et al (2014) Comparison of TiO_2_ nanoparticle and graphene-TiO_2_ nanoparticle composite phototoxicity to *Daphnia magna* and *Oryzias latipes* . Chemosphere 112: 62–69. 25048889 10.1016/j.chemosphere.2014.03.058

[36] Adams LK, Lyon DY, Alvarez PJJ (2006) Comparative eco-toxicity of nanoscale TiO_2_, SiO_2_, and ZnO water suspensions. Water Res 40: 3527–3532. 17011015 10.1016/j.watres.2006.08.004

[37] Li M, Zhu LZ, Lin DH (2011) Toxicity of ZnO nanoparticles to *Escherichia coli*: Mechanism and the influence of medium components. Environ Sci Technol 45: 1977–1983. 21280647 10.1021/es102624t

[38] Li M, Lin DH, Zhu LZ (2013) Effects of water chemistry on the dissolution of ZnO nanoparticles and their toxicity to *Escherichia coli* . Environ Pollut 173: 97–102. 23202638 10.1016/j.envpol.2012.10.026

[39] Long ZF, Ji J, Yang K, Lin DH, Wu FC (2012) Systematic and quantitative investigation of the mechanism of carbon nanotubes’ toxicity toward algae. Environ Sci Technol 46: 8458–8466. 22759191 10.1021/es301802g

[40] Suresh AK, Pelletier DA, Doktycz MJ (2013) Relating nanomaterial properties and microbial toxicity. Nanoscale 5: 463–474. 23203029 10.1039/c2nr32447d

[41] Wang Z, Lee YH, Wu B, Horst A, Kang Y, et al (2010) Anti-microbial activities of aerosolized transition metal oxide nanoparticles. Chemosphere 80: 525–529. 20478610 10.1016/j.chemosphere.2010.04.047

[42] Szczupak AM, Ulfig K, Morawski AW (2011) The application of titanium dioxide for deactivation of bioparticulates: An overview. Catalysis Today 169: 249–257.

[43] Adams CP, Walker KA, Obare SO, Docherty KM (2014) Size-dependent antimicrobial effects of novel palladium nanoparticles. Plos One 9 (1) e89581: 1–12. 10.1371/journal.pone.0085981 PMC3896427 24465824

[44] Musee N, Thwalaa M, Nota N (2011) The antibacterial effects of engineered nanomaterials: implications for wastewater treatment plants. J Environ Monitor 13: 1164–1183. 10.1039/c1em10023h 21505709

[45] Jiang GX, Shen ZY, Niu JF, Bao YP, Chen J, et al (2011) Toxicological assessment of TiO_2_ nanoparticles by recombinant *Escherichia coli* bacteria. J Environ Monitor 13: 42–48. 10.1039/c0em00499e 21127813

[46] Linsebigler AL, Lu GQ, Yates JT (1995) Photocatalysis on TiO_2_ surfaces: Principles, mechanisms, and selected results. Chem Rev 95: 735–758.

[47] Kelly K, Havrilla C, Brady T, Abramo K, Levin E (1998) Oxidative stress in toxicology: established mammalian and emerging piscine model systems. Environ Health Persp 106: 375–384. 10.1289/ehp.98106375 PMC1533135 9637794

[48] Neal AL (2008) What can be inferred from bacteria–nanoparticle interactions about the potential consequences of environmental exposure to nanoparticles? Ecotoxicology 17: 362–371. 18454313 10.1007/s10646-008-0217-x

[49] Dastjerdi R, Montazer M (2010) A review on the applications of inorganic nano-structured materials in the modification of textiles: focus on anti-microbial properties. Colloid Surface B 79: 5–18. 10.1016/j.colsurfb.2010.03.029 20417070

[50] Foster HA, Ditta IB, Varghese S, Steele A (2011) Photocatalytic disinfection using titanium dioxide: spectrum and mechanism of antimicrobial activity. App Microbiol Biot 90: 1847–1868. 10.1007/s00253-011-3213-7 PMC7079867 21523480

[51] Kumar A, Pandey AK, Singh SS, Shanker R, Dhawan A (2011) Engineered ZnO and TiO_2_ nanoparticles induce oxidative stress and DNA damage leading to reduced viability of *Escherichia coli.* . Free Radical Bio Med 51: 1872–1881. 21920432 10.1016/j.freeradbiomed.2011.08.025

[52] Barnes RJ, Molina R, Xu JB, Dobson PJ, Thompson IP (2013) Comparison of TiO_2_ and ZnO nanoparticles for photocatalytic degradation of methylene blue and the correlated inactivation of gram-positive and gram-negative bacteria. J Nanopart Res 15: 1432.

[53] Pagnout C, Jomini S, Dadhwal M, Caillet C, Thomasc F, et al (2012) Role of electrostatic interactions in the toxicity of titanium dioxide nanoparticles toward *Escherichia coli.* . Colloid Surface B 92: 315–321. 10.1016/j.colsurfb.2011.12.012 22218337

[54] Schwegmann H, Ruppert J, Frimmel FH (2013) Influence of the pH-value on the photocatalytic disinfection of bacteria with TiO_2_ - explanation by DLVO and XDLVO theory. Water Res 47: 1503–1511. 23305684 10.1016/j.watres.2012.11.030

[55] Rincon AG, Pulgarin C (2004) Effect of pH, inorganic ions, organic matter and H_2_O_2_ on *E.coli* K12 photocatalytic inactivation by TiO_2_ implications in solar water disinfection. Appl Catal B: Environ 51: 283–302.

